# Overexpression of hypoxia-inducible factor 1 alpha improves immunomodulation by dental mesenchymal stem cells

**DOI:** 10.1186/s13287-017-0659-2

**Published:** 2017-09-29

**Authors:** Victor G. Martinez, Imelda Ontoria-Oviedo, Carolina P. Ricardo, Sian E. Harding, Rosa Sacedon, Alberto Varas, Agustin Zapata, Pilar Sepulveda, Angeles Vicente

**Affiliations:** 1 MRC Laboratory for Molecular Cell Biology, University College London, Gower Street, London, WC1E 6BT UK; 2 Instituto de Investigación Sanitaria La Fe, Regenerative Medicine and Heart Transplantation Unit, Valencia, Spain; 3 National Heart and Lung Institute, Imperial College, London, UK; 4 Department of Cell Biology, Faculty of Medicine, Complutense University, Plaza de Ramón y Cajal, 28040 Madrid, Spain; 5 Department of Cell Biology, Faculty of Biology, Complutense University, Madrid, Spain

**Keywords:** Mesenchymal stem cells (MSCs), Hypoxia-inducible factor 1 alpha subunit (HIF-1 alpha), Immunomodulation, Cell therapy, Dental pulp

## Abstract

**Background:**

Human dental mesenchymal stem cells (MSCs) are considered as highly accessible and attractive MSCs for use in regenerative medicine, yet some of their features are not as well characterized as other MSCs. Hypoxia-preconditioning and hypoxia-inducible factor 1 (HIF-1) alpha overexpression significantly improves MSC therapeutics, but the mechanisms involved are not fully understood. In the present study, we characterize immunomodulatory properties of dental MSCs and determine changes in their ability to modulate adaptive and innate immune populations after HIF-1 alpha overexpression.

**Methods:**

Human dental MSCs were stably transduced with green fluorescent protein (GFP-MSCs) or GFP-HIF-1 alpha lentivirus vectors (HIF-MSCs). A hypoxic-like metabolic profile was confirmed by mitochondrial and glycolysis stress test. Capacity of HIF-MSCs to modulate T-cell activation, dendritic cell differentiation, monocyte migration, and polarizations towards macrophages and natural killer (NK) cell lytic activity was assessed by a number of functional assays in co-cultures. The expression of relevant factors were determined by polymerase chain reaction (PCR) analysis and enzyme-linked immunosorbent assay (ELISA).

**Results:**

While HIF-1 alpha overexpression did not modify the inhibition of T-cell activation by MSCs, HIF-MSCs impaired dendritic cell differentiation more efficiently. In addition, HIF-MSCs showed a tendency to induce higher attraction of monocytes, which differentiate into suppressor macrophages, and exhibited enhanced resistance to NK cell-mediated lysis, which supports the improved therapeutic capacity of HIF-MSCs. HIF-MSCs also displayed a pro-angiogenic profile characterized by increased expression of *CXCL12/SDF1* and *CCL5/RANTES* and complete loss of *CXCL10/IP10* transcription.

**Conclusions:**

Immunomodulation and expression of trophic factors by dental MSCs make them perfect candidates for cell therapy. Overexpression of HIF-1 alpha enhances these features and increases their resistance to allogenic NK cell lysis and, hence, their potential in vivo lifespan. Our results further support the use of HIF-1 alpha-expressing dental MSCs for cell therapy in tissue injury and immune disorders.

**Electronic supplementary material:**

The online version of this article (doi:10.1186/s13287-017-0659-2) contains supplementary material, which is available to authorized users.

## Background

Among different sources of mesenchymal stem cells (MSCs), dental MSCs (dental pulp, periodontal ligament, apical papilla, dental follicle, and gingival tissue) are currently considered as one of the most attractive MSCs for use in engineering tissue and regenerative medicine. Great advantages presented by dental MSCs include accessibility, easy in vitro expansion, low immunogenicity, and the ability to differentiate into mesoderm-derived tissues [1]. A low frequency of in vivo differentiation of transplanted MSCs in experimental models [2–5] indicates that MSCs promote tissue repair through paracrine modulation of surrounding cells. Cell populations normally targeted by MSCs include resident progenitor cells, endothelial cells, and immune cells [6].

Many efforts are currently focused on furthering the therapeutic potential of MSCs. In this regard, we and others have previously demonstrated that hypoxia and, more specifically, overexpression of hypoxia-inducible factor-1 (HIF-1) by genetic engineering potentiates the therapeutic properties of MSCs [7]. Owing to the critical role that the immune response plays in most of the pathologies targeted by MSC therapy, a better understanding of the effects of hypoxia on the immunomodulatory capacity of MSCs is of great importance.

In the present study, we present a comprehensive characterization of the in vitro immunomodulatory properties and expression of immune and trophic factors in dental MSCs. Furthermore, we demonstrate that overexpression of HIF-1 alpha improves several of these features, also enhancing their resistance to natural killer (NK) cell lytic activity.

## Methods

All procedures were approved by the Instituto de Salud Carlos III and Institutional Ethical and Animal Care Committees.

### Vector production, lentiviral transduction, and MSC culture

MSCs from human dental pulp were expanded as described previously [8] and transduced with lentiviral vectors pWP-green fluorescent protein (GFP-MSCs) or pWP-HIF-GFP (HIF-MSCs) as previously described [7]. Transduction efficiency was evaluated by flow cytometry (Coulter EPICS XL flow cytometer; Beckman Coulter) to determine the percentage of GFP-positive cells. The percentages of infection obtained were normally around 90%. Human dental pulp MSCs (*n* = 3; Inbiobank) were cultured in Dulbecco’s modified Eagle’s medium (DMEM) low glucose (Sigma-Aldrich, Spain) supplemented with 10% heat-inactivated fetal calf serum (FCS; Gibco, Life Technologies, Thermo Fisher Scientific). Cells were grown until confluence and subsequently subcultured up to 12 passages. For stimulation assays, 5 × 10^4^ MSCs were seeded in six-well flat-bottom culture plates and, after 3 days, media were replenished and supplemented or not with 10 ng/ml recombinant human interferon (rhIFN)-gamma (Invitrogen). After 15 h, cells were lysed for subsequent analysis by quantitative polymerase chain reaction (qPCR).

### Seahorse extracellular metabolic-flux assays

The Seahorse extracellular flux analyzer XFp (Seahorse Bioscience) was used to perform the mitochondrial and glycolysis stress tests. Detailed conditions are given in Additional file 1. In both assays, total protein per well was quantified with the BCA Protein Assay Kit for normalization.

#### Mitochondrial stress test

Four basal oxygen consumption rate (OCR) measurements were taken. Subsequently, mitochondrial membrane modulators oligomycin (1 μM), followed by carbonyl cyanide p-trifluoromethoxy-phenylhydrazone (FCCP; 1 μM) and finally antimycin A/rotenone (both 1 μM) were injected (four measuring cycles each). Basal respiration, ATP production, maximal respiration, spare respiratory capacity, and proton leak were defined by the differences between the average measurements 4–16, 4–8, 12–16, 4–9, and 8–16, respectively. Non-mitochondrial respiration was defined by the average measurement 16.

#### Glycolysis stress test

Extracellular acidification rate (ECAR) measurements were taken. Subsequently, glucose (10 mM), followed by oligomycin (1 μM) and 2-deoxy-glucose (2-DG; 100 mM) were injected (four measuring cycles each). Basal glycolysis, glycolytic capacity, and glycolytic reserve were calculated by the differences between the average measurements 4–8, 12–16, and 8–12, respectively. Non-glycolytic acidification was defined by the average measurement 16.

### Microarray assays

Human MSCs from dental pulp cultured under normoxia, hypoxia, and lentivirally transduced with GFP-HIF-1 alpha or GFP empty vector (*n* = 3 different donors) were collected and 800 ng total RNA was extracted. RNA purity and concentration were evaluated spectrophotometrically using a NanoDrop ND-2000 (ThermoFisher). A260/230 and A260/280 ratios were mainly used, but the full spectra were reviewed to assess the presence of contaminants: peptides, phenols, aromatic compounds, or carbohydrates, and proteins. The quality and integrity of total RNA from the cell cultures were assessed by a microfluidics-based platform Agilent 2100 Bioanalyzer (Agilent Technologies, Santa Clara) with the Agilent RNA 6000 Nano Kit for RIN calculation. Electropherograms were visualized with the Agilent 2100 Expert software including data collection, peak detection, and interpretation of the different profiles.

RNA expression levels were measured by microarrays. All control and sample RNAs tested were selected according to integrity criteria. Expression profile was determined using the PrimeView Affymetrix platform including all annotated genes based in the NCBI Human Genome version 37 (GRCh37). This version contains 48,658 probe sets including 419 UniGene probe sets not covered by RefSeq probe sets.

Total RNA (250 ng) was retrotranscribed to cDNA from polyA tail, double-stranded and transcribed into cRNA. Labeling and fragmentation of this cRNA was performed following the manufacturer’s procedures. The GeneChip® Scanner 3000 7G System and reagents from Affymetrix were used to hybridize, wash, stain, and scan the arrays. Hybridization was performed by the microarray core facility from IIS La Fe.

### Microarray data analysis

Differential gene expression was measured using fold-change. Results were considered to be significant with a 1.5-fold induction. Gene ontology (GO) analysis was carried out with the Consensuspathdb software tool (http://cpdb.molgen.mpg.de/) [9] and used to identify the biological pathways associated with the mRNAs differentially expressed in HIF-MSCs vs GFP-MSCs, and in MSC cultured under hypoxia versus normoxia conditions for comparison.

### Isolation and culture of human blood cells

Buffy coats of healthy donors were obtained after informed consent (Centro de Transfusión de la Comunidad de Madrid, Spain) and peripheral blood mononuclear cells (PBMCs) were isolated by density gradient centrifugation with Lymphocyte Isolation Solution (Rafer). Monocytes and NK cells were isolated by positive and negative magnetic separation (MiltenyiBiotec). Non-adherent T lymphocyte-enriched cell suspensions were obtained by nylon wool purification. The percentage of CD3^+^ cells was always above 85%.

CD14^+^ cells (5 × 10^5^) were cultured in six-well flat-bottom culture plates for 6 days in 50% DMEM/10% FCS and 50% complete medium, consisting of RPMI (Lonza) supplemented with 10% FCS, 1 mM pyruvate, 2 mM glutamine, 100 U/ml penicillin and 100 μg/ml streptomycin (all from Sigma-Aldrich, Spain), plus 20 ng/ml recombinant human granulocyte macrophage-colony stimulating factor (rhGM-CSF) and 20 ng/ml recombinant human interleukin (rhIL)-4 (Invitrogen) to generate immature monocyte-derived dendritic cells (MoDCs), or 5 ng/ml rhGM-CSF to generate monocyte-derived macrophages (MDMs). In MoDC cultures, half of the medium was replaced by fresh medium and rhGM-CSF/rhIL-4 on days 2 and 4 of culture. In MDM cultures, an additional 5 ng/ml rhGM-CSF was added every 2 days and 10 ng/ml of lipopolysaccharide (LPS; Invitrogen) was added for an additional 24 h of culture. When indicated, 5 × 10^4^ MSCs (MSC:monocyte ratio 1:10) were seeded in six-well flat-bottom culture plates, monocytes were added the day after, and co-cultures were performed as stated above.

Freshly isolated NK cells were cultured in 24-well flat-bottom culture plates for 36 h in 50% DMEM/10% FCS and 50% complete media supplemented with 10 ng/ml rhIL-12 and 10 ng/ml rhIL-15 (MiltenyiBiotec). As a negative control, NK cells were cultured in media alone. MSC-NK cell co-cultures were performed at MSC:NK cell ratios of 1:10 and 1:20, seeding MSCs the day before and keeping a number of 3 × 10^4^ MSCs.

For T-cell activation, enriched T-cell suspensions (3 × 10^5^) were cultured in 24-well flat-bottom culture plates for 4 days with immobilized anti-human CD3 (HIT3a; 10 μg/ml) and soluble anti-human CD28 (CD28.2; 1 μg/ml) monoclonal antibodies (BioLegend). As a negative control, T cell-enriched cells were cultured in media alone. MSC-T cell co-cultures were performed at a MSC:T cell-enriched ratio of 1:10, seeding MSCs the day before.

### Flow cytometry

See Additional file 1 (Table S1) for a list of antibodies used. Immunofluorescence staining was performed as described previously [10]. For intracellular staining, brefeldin A (BioLegend) was added for the final 4 h of culture and cells were treated with the Fixation/Permeabilization Solution Kit (BD Biosciences). All flow cytometry analyses were conducted in a FACSCalibur flow cytometer (BD Biosciences) from the Centro de Citometría y Microscopía de Fluorescencia, Complutense University of Madrid, Spain.

### Proliferation assays

Before culture, T lymphocyte-enriched cell suspensions were labeled with 5 μM carboxyfluoroscein succinimidyl ester (CFSE; BioLegend). Proliferation of T cells (CD3^+^/CD4^+^ and CD3^+^/CD8^+^) was determined after 4 days by the CFSE dilution method. T cells were gated according to forward and side scatter.

### Apoptosis assays

The proportion of apoptotic MSCs was determined by incorporation of propidium iodide. MSC death was calculated as the percentage of CD90^+^/propidium iodide^+^ cells.

### Migration assays

MSCs (25 × 10^3^) were seeded in 24-well flat-bottom culture plates. The next day, culture media with 10 ng/ml rhIFN-gamma was added and 6.5-mm insert, 8.0-μm polycarbonate membrane Transwell permeable supports (Corning Life Sciences) were mounted. PBMCs (10^6^) were added into the inserts. Transwell cultures without MSCs in the lower chamber were used as controls. Cells were cultured for 14 h and migrated cells (present at the lower chamber) were collected and stained for CD14. The number of CD14^+^ cells were counted on a FACSCalibur flow cytometer and gated according to forward/side scatter and lack of CD90 expression. Monocyte migration (MM) was calculated as follows:

$$ \mathrm{MM}=\frac{\left({\mathrm{N}}^{{}^{\circ}}\ \mathrm{CD}{14}^{+}\ \mathrm{cells}\  \mathrm{with}\  \mathrm{MSCs}\right)-\left({\mathrm{N}}^{{}^{\circ}}\ \mathrm{CD}{14}^{+}\ \mathrm{cells}\  \mathrm{with}\mathrm{out}\  \mathrm{MSCs}\right)}{{\mathrm{N}}^{{}^{\circ}}\ \mathrm{CD}{14}^{+}\ \mathrm{cells}\  \mathrm{with}\mathrm{out}\  \mathrm{MSCs}}\times 100 $$

### PCR analysis

RNA isolation was performed using the Absolutely RNA Microprep kit (Stratagene Cloning Systems, Agilent Technologies, Santa Clara, CA, USA), including DNase I digestion. Total cDNA was synthesized by the High Capacity cDNA Reverse Transcription Kit (Applied Biosystems, Thermo Fisher Scientific). Taq-man assays (Applied Biosystems) employed for real-time PCR are shown in Additional file 1 (Table S2). All PCR reactions were set in duplicates using the TaqMan Gene Expression Master Mix (Applied Biosystems). Amplifications, detections, and analyses were performed in a 7.900HT Fast Real-time PCR System (Centro de Genómica, Complutense University, Madrid, Spain). The delta CT method was used for normalization to GNB2L1 mRNA.

### Cytokine measurements

Levels of IFN-gamma (R&D Systems), tumor necrosis factor (TNF)-alpha and IL-10 in culture supernatants were assayed by enzyme-linked immunosorbent assay (ELISA; BioLegend). Production of CCL2/MCP-1 was measured by cytometric bead array (CBA; BD Biosciences).

### Statistical analysis

Student *t* test was used for comparison of two sets of data and one-way analysis of variance (ANOVA) for multiple variables. Values of *p* ≤ 0.05 were considered to be statistically significant.

## Results

### Efficient transduction of functionally active HIF-1 alpha in dental MSCs

**Fig. 1:**
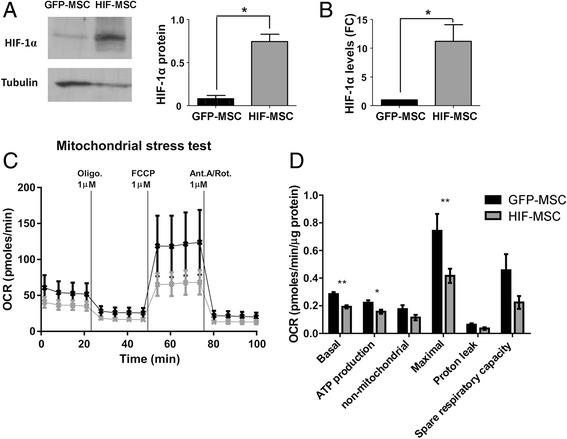
Overexpression of HIF-1 alpha and mitochondrial stress test in MSCs. **a** Representative Western blot of hypoxia-inducible factor-1 (*HIF-1*) alpha in mesenchymal stem cells (*MSCs*) and HIF-MSCs. Loading control was performed with tubulin and levels of HIF-1 alpha protein expression were quantified by densitometry (*right panel*). **b** Expression of the HIF-1 alpha mRNA determined by quantitative PCR. GNB2L1 was used as endogenous control. Date represent expression in HIF-MSCs relative to MSCs control (fold change; *FC*). Quantification of four independent experiments; data are represented as mean ± SE (Student *t* test, *p ≤ 0.05). **c** Oxygen comsumption rate (*OCR*) response to oligomycin (*Oligo.*; 1 μM), carbonyl cyanide p-trifluoromethoxy-phenylhydrazone (*FCCP*; 1 μM), and antimycin A/rotenone (*Ant.A/Rot.*; both 1 μM) injections. HIF-MSCs (*gray line*) showed a lower OCR than MSCs (*black line*). **d** Mitochondrial substrate utilization in HIF-MSCs (*gray*) and MSCs (*black*) measured in terms of basal respiration, ATP production, non-mitochondrial respiration, maximal respiration, proton leak, and spare respiratory capacity. Data were normalized against the total amount of protein. Data are represented as mean ± SE of six independent experiments (Student *t* test, *p ≤ 0.05, **p ≤ 0.01). *GFP* green fluorescent protein

MSCs from dental pulp were expanded and transduced with the lentiviral vectors pWP-GFP (GFP-MSCs) or pWP-HIF-GFP (HIF-MSCs). Efficient transduction of MSCs was confirmed by Western blot and qPCR, showing increased levels of HIF-1 alpha protein and mRNA in HIF-MSCs (Fig. 1a and b).

To investigate the impact of HIF-1 alpha overexpression on the metabolic state of MSCs, we performed mitochondrial and glycolysis stress tests to compare the OCR and glycolysis, respectively, between HIF-MSCs with control GFP-MSCs. HIF-MSCs presented lower basal respiration, ATP production, and maximal respiration rates as assessed by injections of 1 μM oligomicin, 1 μM FCCP, and 1 μM antimycin A/rotenone (Fig. 1c and d). Additionally, HIF-MSCs tended to show a higher ECAR upon injection of 10 mM glucose (glycolysis) and 1 μM oigomycin (glycolytic capacity), indicating a possible effect of HIF-1 alpha in enhancing glycolysis (Additional file 1: Figure S1).

We further evaluated gene expression changes induced by HIF-1 alpha by microarray analysis (Additional file 1: Table S3). Gene Ontology analysis of genes upregulated in HIF-MSCs versus GFP-MSCs revealed significant enrichment of genes involved in biological processes associated with hypoxia. Of note, similar results were obtained when comparing hypoxia- versus normoxia-cultured MSCs. These results indicate that overexpression of HIF-1 alpha recapitulates, at least in part, transcriptional changes induced by hypoxia in MSCs. Altogether, protein and mRNA levels and metabolic and transcriptomic changes demonstrate that HIF-1 alpha is functionally overexpressed in dental HIF-MSCs.

### Effects of HIF-1 alpha overexpression in the modulation of the adaptive immune response by dental MSCs

**Fig. 2:**
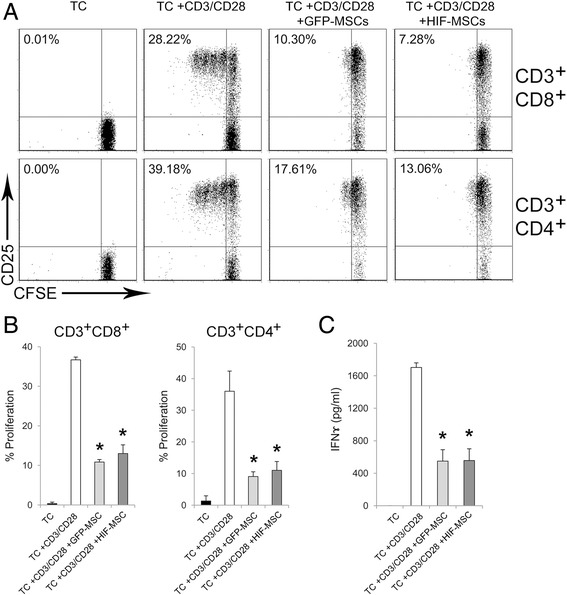
HIF-1 alpha overexpression does not modify the ability of MSCs to inhibit T-cell activation. T cell-enriched peripheral blood cells were stained with carboxyfluoroscein succinimidyl ester (*CFSE*) and activated with anti-CD3/anti-CD28 monoclonal antibodies in the presence or absence of mesenchymal stem cells (*MSCs*; *light grey*) and hypoxia-inducible factor (*HIF*)-MSCs (*dark grey*). T cells (*TC*) were cultured in media alone as a negative control. After 4 days, expression of CD25 and proliferation of T-cell subsets was determined by flow cytometry. A representative experiment (**a**) and the mean ± SD of three independent experiments (**b**) are shown. T cells were gated according to forward/side scatter characteristics and expression of CD3, CD4, and CD8. **c** Supernatants of cultures were collected after 4 days and assayed for interferon (*IFN*)-gamma production. Data are presented as mean ± SD of three independent experiments. One-way ANOVA, *p ≤ 0.05, GFP-MSCs and HIF-MSCs versus no MSCs. *GFP* green fluorescent protein

T cells and dendritic cells (DCs) are main players in adaptive immunity, with T cells acting as main effectors when a deleterious inflammatory response is developed. In addition, impairment of the T-cell response is a well-established feature of MSCs [11, 12]. As a first approach, we investigated the impact of HIF-1 alpha expression on the ability of MSCs to inhibit TCR-triggered activation of T cells. When T cells were activated in the presence of MSCs, proliferation of both CD4^+^ and CD8^+^ T cells was dramatically reduced, regardless of HIF-1 alpha overexpression by MSCs (Fig. 2a and b). As expected, levels of IFN-gamma secreted by activated T cells were severely reduced in the presence of both GFP-MSCs and HIF-MSCs (Fig. 2c).

**Fig. 3:**
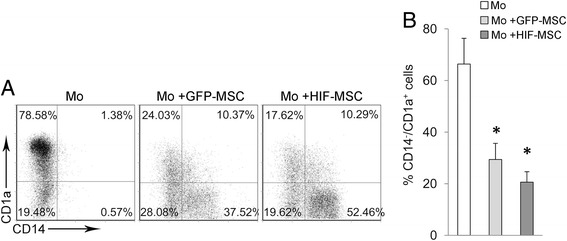
Inhibition of dendritic cell differentiation by dental MSCs. Monocytes were differentiated towards dendritic cells with rhGM-CSF and rhIL-4 alone (*Mo*) or in the presence of control green fluorescent protein (*GFP*)-mesenchymal stem cells (*MSCs*; *light grey*) and hypoxia-inducible factor (*HIF*)-MSCs (*dark grey*). After 7 days, cells were harvested, and the expression of CD14 and CD1a was analyzed by flow cytometry. A representative experiment (**a**) and mean ± SD of four independent experiments (**b**) are shown. MSCs were excluded from the analysis by CD90 expression. One-way ANOVA, *p ≤ 0.05, GFP-MSCs and HIF-MSCs versus no MSCs

We next tested whether HIF-1 alpha overexpression could improve the ability of MSCs to dampen DC differentiation. Dental MSCs negatively affected the generation of CD14^–^CD1a^+^ MoDCs from monocytes. Importantly, the inhibition of MoDC differentiation showed a higher trend in HIF-MSC co-cultures, with a constant reduction in the percentage of CD14^–^CD1a^+^ MoDCs observed in control co-cultures (Fig. 3a and b).

### Overexpression of HIF-1 alpha by MSCs potentiates recruitment of monocytes which acquire immunosuppressive properties

**Fig. 4:**
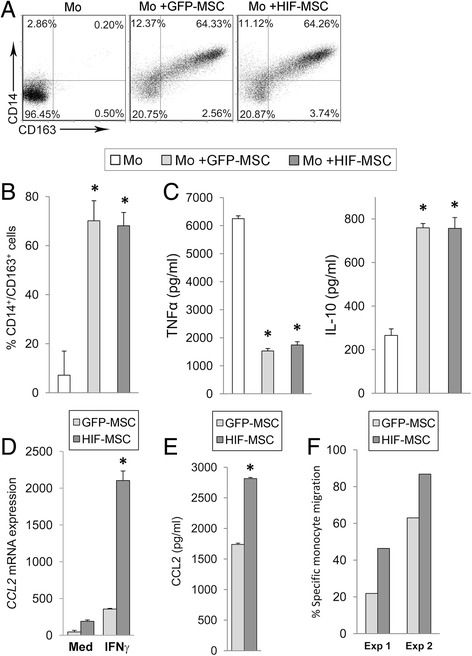
Monocyte differentiation and attraction in the presence of dental MSCs. Monocytes alone (*Mo*) or in the presence of green fluorescent protein (*GFP*)-mesenchymal stem cells (*MSCs*; *light grey*) and hypoxia-inducible factor (*HIF*)-MSCs (*dark grey*) were cultured with rhGM-CSF to induce macrophage differentiation. After 7 days, the expression of CD14 and CD163 was determined by flow cytometry. A representative experiment (**a**) and mean ± SD of three independent experiments (**b**) are shown. MSCs were excluded from the analysis by CD90 expression. **c** After 7 days of culture, LPS was added for 24 h and supernatants were assayed for tumor necrosis factor (*TNF*) alpha and interleukin (*IL*)-10 production. Data are presented as mean ± SD of three independent experiments. One-way ANOVA, *p ≤ 0.05, GFP-MSCs and HIF-MSCs versus no MSCs. **d**, **e** MSCs were cultured until 80% confluence, then fresh medium alone (*Med*) or medium supplemented with IFN-gamma (*IFN*γ) was added. After 15 h, cells and supernatants were harvested. Transcript (**d**) and protein levels (**e**) for CCL2 were determined by quantitative PCR and CBA, respectively. GNB2L1 was used as endogenous control. Mean ± SD of four samples pooled from two independent experiments is shown. Student *t* test, *p ≤ 0.05, MSCs vs HIF-MSCs). **f** Transwell (8 μm) cultures were performed as stated in the Methods section. After 14 h, migrated cells (present at the lower chamber) were collected and stained for CD14. MSCs were excluded from the analysis by CD90 expression and monocytes were gated according to forward/side scatter characteristics and expression of CD14, and counted in a FACSCalibur flow cytometer

The generation of pro-inflammatory macrophages from blood monocytes in response to environmental factors is a key phenomenon in the development of inflammation and tissue damage [13]. Our results show that the majority of monocytes cultured with both types of MSCs exhibited a CD14^high^/CD163^high^ phenotype (Fig. 4a and b). This phenotype has been previously associated with suppressor or M2 features in macrophages [14]. In line with this, we found that the production of TNF-alpha was severely impaired by the presence of both GFP-MSCs and HIF-MSCs while IL-10 levels were remarkably increased in these co-cultures (Fig. 4c).

From a therapeutic point of view these results suggest that the recruitment of monocytes to the site of inflammation could be advantageous in patients transplanted with dental MSCs. In this regard, CCL2/MCP-1 is the main chemokine driving monocyte extravasation. We found that HIF-MSCs exhibited augmented transcription of *CCL2/MCP-1* and that this difference was further enhanced in response to IFN-gamma (Fig. 4d). Coherently, secretion of CCL2/MCP-1 was significantly higher in IFN-gamma-stimulated HIF-MSCs (Fig. 4e), while basal levels of this chemokine were hardly detected (data not shown). Migration assays in the presence of IFN-gamma showed that the higher production of CCL2/MCP-1 observed in HIF-MSCs was accompanied by enhanced migration of monocytes (Fig. 4f).

### HIF-1 alpha overexpression confers resistance to NK cell-mediated lysis

**Fig. 5:**
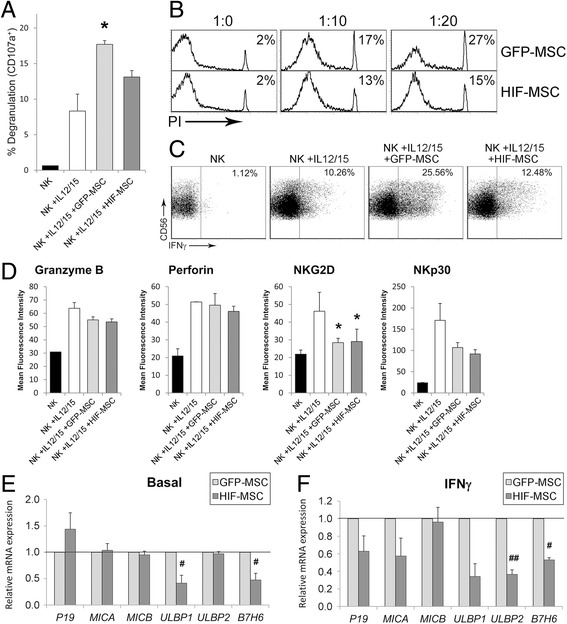
HIF-1 overexpression in MSCs confers resistance to NK cell-mediated lysis. Natural killer (*NK*) cells were cultured in media alone (*black bars*) or with rhIL-12 and rhIL-15 in the absence (*white bars*) or presence of green fluorescent protein (*GFP*)-mesenchymal stem cells (*MSCs*; *light grey*) and hypoxia-inducible factor (*HIF*)-MSCs (*dark grey*). After 36 h of culture, expression of CD107a (**a**), intracellular interferon (*IFN*)-gamma (**c**) and the indicated molecules (**d**) was analyzed in NK cells by flow cytometry. MSCs were excluded from the analysis by CD90 expression. **b** MSC-NK cell co-cultures were performed at the indicated MSC:NK cell ratios in presence of rhIL-12 and rhIL-15. After 36 h late apoptosis of MSCs was determined by CD90 expression and propidium iodide (*PI*) incorporation. A representative experiment (**b** and **c**) and mean ± SD of three independent experiments (**a** and **d**) are shown. One-way ANOVA, *p ≤ 0.05, GFP-MSCs and HIF-MSCs versus no MSCs. **e**, **f** MSCs were cultured until 80% confluence was reached, and then culture medium was replaced by fresh medium alone (**e**) or supplemented with IFN-gamma (**f**). After 15 h, cells were harvested and expression of the indicated genes was determined by qPCR. GNB2L1 was used as endogenous control. Bars represent expression in HIF-MSCs relative to GFP-MSC controls. Mean ± SD of six to eight samples pooled from at least three independent experiments is shown. Student *t* test, ^#^ *p* ≤ 0.05, ^##^ *p* ≤ 0.005, GFP-MSCs versus HIF-MSCs. *IL* interleukin

Poor mid-term persistence of transplanted MSCs limits the therapeutic impact of MSCs, most notably in myocardial infarction cell therapy [15]. NK cells have been postulated as key mediators in this process since they efficiently lyse autologous and allogeneic MSCs, participating in the clearance of these cells after transplantation, also accompanied by IFN-gamma secretion [16]. Consistent with the literature, we found that degranulation of activated NK cells was significantly increased in the presence of control GFP-MSCs (Fig. 5a). Nevertheless, in HIF-MSC co-cultures, increased NK cell degranulation was impaired. In accordance with this, HIF-MSCs exhibited a marked resistance to NK cell-mediated death (Fig. 5b). Also importantly, increased IFN-gamma production by NK cells was strongly reduced by HIF-MSCs (Fig. 5c).

Interestingly, increased resistance of HIF-MSCs to NK cell-mediated lysis was not associated with differential modulation of the NK cell effector molecules granzyme B and perforin (Fig. 5d). Activation of the receptors Natural Killer Group 2D (NKG2D) and NKp30 has been shown to be critical for optimal lysis of MSCs by activated NK cells [16]. Nevertheless, we found no differences in the expression of NKG2D and NKp30 between NK cells activated in the presence of GFP-MSCs or HIF-MSCs (Fig. 5d). On the other hand, analysis of the expression by MSCs of the ligands for these NK cell-activating receptors revealed a reduction in the basal expression of *ULBP1* and *B7H6* in HIF-MSCs (Fig. 5e). These dissimilarities were strengthened after stimulation with IFN-gamma, a pro-inflammatory factor highly produced by activated NK cells, again with a reduced expression of *ULBP1*, *B7H6*, and *ULBP2* on HIF-MSCs (Fig. 5f).

### Enhanced immunosuppressive and pro-angiogenic features in HIF-1 alpha expressing MSCs

**Fig. 6:**
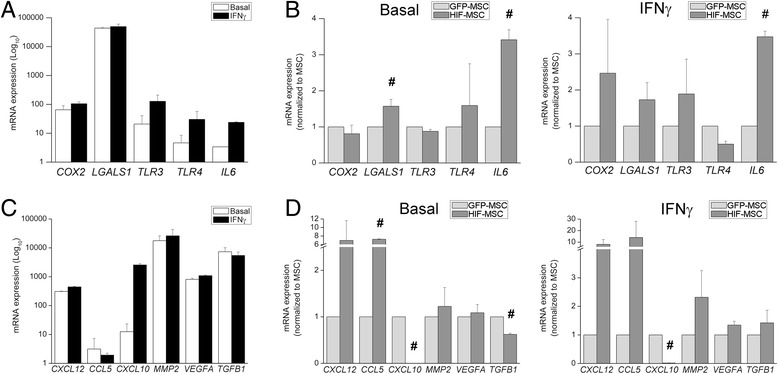
Immunomodulatory and pro-angiogenic profile induced by HIF-1 alpha expression in MSCs. Mesenchymal stem cells (*MSCs*) were cultured until 80% confluence was reached, and then culture medium was replaced by fresh medium alone (*Basal*) or supplemented with IFN-gamma (*IFN*γ). After 15 h, cells were harvested and expression of the indicated genes was determined by qPCR. GNB2L1 was used as endogenous control. **a**, **c** Expression of the indicated genes in control green fluorescent protein (*GFP*)-MSCs. Note that a logarithmic scale is used. **b**, **d** Expression in hypoxia-inducible factor (*HIF*)-MSCs relative to MSC controls. Mean ± SD of six to eight samples pooled from at least three independent experiments is shown. Student *t* test, ^#^ *p* ≤ 0.05, MSCs vs HIF-MSCs

Next, we analyzed the expression of relevant factors regulating inflammation and tissue remodeling. Figure 6a represents the expression of immunomodulatory factors in control dental GFP-MSCs at basal levels and after IFN-gamma stimulation. As shown in the figure, IFN-gamma induced in GFP-MSCs the expression of *TLR3*, *TLR4*, *IL6*, and, to a lesser extent, *COX2*. On the contrary, *LGALS1*, encoding for galectin 1, was greatly expressed by dental GFP-MSCs but not induced after IFN-gamma stimulation (Fig. 6a). Comparative analysis showed enhanced basal expression of the immunosuppressive molecules galectin 1 and IL-6 in HIF-MSCs (Fig. 6b, left panel). In an inflammatory context, mimicked by IFN-gamma stimulation, HIF-MSCs maintained a higher expression of *IL6* (Fig. 6b, right panel).

We also found that dental GFP-MSCs differentially expressed a number of pro-angiogenic and trophic factors of which only *CXCL10* showed strong induction after IFN-gamma treatment (Fig. 6c). Expression of *CXCL12* and *CCL5* was higher in HIF-MSCs both at basal levels and after the addition of IFN-gamma, while the basal transcription of the pro-fibrotic factor *TGFB1* was reduced (Fig. 6d, left and right panels). Remarkably, overexpression of HIF-1 alpha caused a complete loss of *CXCL10* transcription in HIF-MSCs under basal and inflammatory conditions.

## Discussion

Dental MSC-based cell therapy is currently under extensive research over a wide range of pathologies in which inflammation plays a key role. These include wound healing, spinal cord injury, and periodontitis [2, 17, 18]. While suppressor activities through T cell and macrophage responses have been previously described [19], the role of dental MSCs cells in multiple immune interactions remains elusive.

Our results show that inhibition of CD4^+^ and CD8^+^ T-cell responses to TCR stimulation by dental MSCs is independent of HIF-1 alpha expression. This might be correlated with the lack of differences in the expression of COX2/PGE2, IL-10, and IDO (Fig. 5 and data not shown), main factors driving T-cell inhibition by dental MSCs [20, 21]. Recently, it was demonstrated that inhibition of the proliferation of PBMCs by gingival MSCs is increased after hypoxia preconditioning, with the participation of IL-10 and FasL [22]. Since total PBMCs were used in these experiments, it is difficult to know whether T cells were directly targeted by hypoxic gingival MSCs. Accordingly, inhibition of T-cell proliferation by adipose tissue-derived MSCs is not altered under hypoxic conditions [23].

On the other hand, we found that HIF-1 alpha overexpression induces higher expression *IL6*, a known negative regulator of DC differentiation and function [24, 25]. Enhanced expression of *IL6* has also been shown in hypoxia-conditioned MSCs from bone marrow [26]. This could explain that impairment of DC differentiation by dental MSCs shows certain enhancement when MSCs express HIF-1 alpha.

We also discovered that HIF-MSCs produce higher levels of the chemokine *CCL2/MCP1* under inflammatory conditions, showing as well a tendency to promote greater attraction of monocytes. This is in agreement with recent data showing that conditioned medium from hypoxic bone marrow-derived MSCs enhances monocyte migration and recruitment of F4/80^+^ macrophages during wound healing [26]. Recent reports show that dental MSCs induce suppressor macrophage differentiation with the participation of different factors, including IL-6 and CCL2/MCP-1 [18, 27]. Furthermore, we present here that monocytes cultured with dental MSCs exhibit high IL-10 and low TNF-alpha production after LPS/TLR-4 stimulation. It is important to note that TLR-4 agonists are normally present after tissue damage [28]. Owing to the beneficial role normally attributed to anti-inflammatory macrophages [29], we propose that expression of HIF-1 alpha by MSCs would be advantageous for the resolution of inflammation through enhanced recruitment and differentiation of suppressor macrophages.

NK cell-mediated lysis of MSCs might be linked to the poor mid-term persistence of transplanted MSCs observed in vivo [15, 16, 30]. Here, we confirm these observations showing that NK cells are capable of killing dental MSCs, along with increased production of IFN-gamma. Nevertheless, HIF-MSCs exhibit increased resistance to NK cell-mediated lysis as shown by reduced MSC cell death, NK cell degranulation, and IFN-gamma production. Efficient NK cell-mediated lysis requires binding of the activating receptors expressed on NK cells to their natural ligands, expressed in MSCs [16]. Consistently, we show that HIF-MSCs exhibit a significantly reduced expression of the NK cell-activating receptor ligands ULBP1 and B7H6 [31]. Interestingly, this mechanism is frequently exploited by tumor cells [32–34]. These results are coherent with the increased engraftment and survival of hypoxia-conditioned MSCs after transplantation [35, 36]. Considering that poor mid-term persistence of transplanted MSCs is a limiting factor in the therapeutic potential of MSCs [15], we here provide evidence that supports HIF-1 alpha expression as a potential useful approach to overcoming this limitation.

MSCs may exert tissue repair and regeneration through paracrine factors, acting on surrounding cells and directing cytoprotection and neovascularization [6]. We have found that HIF-MSCs exhibit enhanced expression of *CXCL12/SDF1* and *CCL5/RANTES*, factors widely known by their pro-angiogenic actions [37, 38]. Strikingly, our results demonstrate that HIF-1 alpha expression in MSCs completely abrogates *CXCL10/IP10* expression. This factor can limit new vessel growth by inhibiting endothelial cell migration, and can induce involution of new vessels [39]. Similarly, enhanced angiogenic potential of hypoxia-conditioned MSCs has been shown in two different experimental models for ischemia [35, 40].

## Conclusions

Hypoxia preconditioning of MSCs significantly improves the therapeutic effects of MSCs. Furthermore, this procedure has been suggested for standardized protocols of MSC preparation for clinical applications [41]. The present work demonstrates that HIF-1 alpha overexpression confers to MSCs enhanced paracrine capacities which might participate in the previously described benefits of HIF-MSC therapy after myocardial infarction [7]. Furthermore, HIF-MSCs exhibit increased resistance to NK cell-mediated lysis, which will help to prolong their lifespan in the host. Although further experiments using immunocompetent animals are required to interrogate in vivo the mechanism described in the present study, we believe that dental MSCs and genetic engineering to express HIF-1 alpha are powerful tools for furthering the therapeutic potential of cell therapy.

## Additional file

Additional file 1: Detailed methods: lentiviral production and transduction, metabolic assays, and list of reagents for flow cytometry and PCR. **Table S1.** List of antibodies used for flow cytometry. **Table S2.** List of Taq-man assays used for qPCR. **Table S3.** Analysis of GO pathways shared in HIF-MSC vs GFP-MSC and MSC hypoxia vs MSC normoxia. **Figure S1.** Results from glycolytic activity of MSCs. (DOCX 130 kb)

## Abbreviationss

CFSE: Carboxyfluoroscein succinimidyl ester
DC: Dendritic cell
DMEM: Dulbecco’s modified Eagle’s medium
ECAR: Extracellular acidification rate
FCCP: Carbonyl cyanide p-trifluoromethoxy-phenylhydrazone
FCS: Fetal calf serum
GFP: Green fluorescent protein
GM-CSF: Granulocyte macrophage-colony stimulating factor
HIF-1: Hypoxia-inducible factor-1
IFN: Interferon
IL: Interleukin
LPS: Lipopolysaccharide
MDM: Monocyte-derived macrophage
MoDC: Monocyte-derived dendritic cell
MSC: Mesenchymal stem cell
NK: Natural killer
OCR: Oxygen consumption rate
PBMC: Peripheral blood mononuclear cell
qPCR: Quantitative polymerase chain reaction
rh: Recombinant human
TNF: Tumor necrosis factor
