# Supplementary material for: Overexpression of hypoxia-inducible factor 1 alpha improves immunomodulation by dental mesenchymal stem cells
Source: Stem Cell Res Ther. 2017 Sep 29;8:208. doi: 10.1186/s13287-017-0659-2 (PMC5622468; doi:10.1186/s13287-017-0659-2)
Supplement: Additional file 1: — Detailed methods: lentiviral production and transduction, metabolic assays, and list of reagents for flow cytometry and PCR. Table S1. List of antibodies used for flow cytometry. Table S2. List of Taq-man assays used for qPCR. Table S3. Analysis of GO pathways shared in HIF-MSC vs GFP-MSC and MSC hypoxia vs MSC normoxia. Figure S1. Results from glycolytic activity of MSCs. (DOCX 130 kb) [file 13287_2017_659_MOESM1_ESM.docx]

**Overexpression of hypoxia-inducible factor 1 alpha improves immunomodulation by dental mesenchymal stem cells.**

Martínez VG.^1^, Ontoria-Oviedo I.^2^, Ricardo CP.^3^, Harding SE.^3^, Sacedón R.^1^, Varas A.^1^, Zapata A.^4^, Sepúlveda P.^2^, Vicente A.^1^

^1^Department of Cell Biology, Faculty of Medicine, Complutense University, Madrid, Spain.

^2^Instituto de Investigación Sanitaria La Fe, Regenerative Medicine and Heart Transplantation Unit, Valencia, Spain.

^3^National Heart and Lung Institute, Imperial College, London, UK.

^4^Department of Cell Biology, Faculty of Biology, Complutense University, Madrid, Spain.

**Additional file 1: Supplementary Material**

**Detailed methods**

**Vector production**

All the plasmids used were from Addgene (Cambridge, MA, USA). 6x10^6^ 293T packaging cells were transiently transfected with pCMV-VSV-G (1.5 µg, envelope vector), psPAX2 (3 µg, packaging vector) and pWP-GFP or pWP-HIF-GFP (5 µg, transfer vectors). 50 µl of 2.5 M CaCl2 was added to the plasmid mix and then 500 µl of 2xHEPES-buffer while vortexing. Mixture was incubated at room temperature for 30 min and then added to the 293T cells for 7 h. media was removed and replaced by fresh medio (DMEM high glucose and 10% FBS). Lentiviral particles were collected from the supernantant at 48 h post-transfection, supernatants were filtered through a 0.45 µm filter and lentivirus were concentrated by one round of ultracentrifugation at 4 ºC during 1h 30 minutes at 120000 g using a swinging bucket rotor. Lentiviral pellet was resuspended in 90 µl of PBS + 2% BSA.

**Lentiviral transduction**

3x10^5^ MSCs (passage 5-8) were incubated with corresponding lentivirus previously obtained for 8 h and after the media was replaced by fresh media, this procedure was repeated at least two times. 3 days after the transduction efficiency was evaluated by flow cytometry (Coulter EPICS XL flow cytometer; Beckman Coulter) to determine the percentage of GFP-positive cells. Percentage of infection obtained were normally around 90%.

**Expression of HIF-1 alpha by western blotting**

MSCs and HIF-MSCs were lysed with RIPA-buffer (1.5M NaCl, 10% Nonidet P-40, 500mM Tris-HCl, 5% deoxycholate and 1% SDS, pH 8) supplemented with protease inhibitors (Roche). Cells were disrupted by freeze-thaw cycles and centrifuged at 12,000*g* for 15 min at 4ºC. Total protein was quantified with the Pierce™ BCA Protein Assay Kit (Thermo Scientific). Samples were separated in SDS-PAGE and fractions were detected with anti-HIF1α (H1alpha67, 1:200; Abcam). α-Tubulin was used to normalize loading. Immunoblots were digitized and quantified with ImageJ.

**Oxygen Consumption Measurements**

The Seahorse extracellular flux analyzer XFp (Seahorse Bioscience, Billerica, MA, USA) was used to assess mitochondrial substrate utilization in MSCs as previously described [1]. Briefly, MSC and MSC-HIF were seeded on Seahorse XFp Cell Culture Miniplates at 7500 cells per well, and allowed to attach and proliferate in a 5% CO2, 21% O2 incubator at 37 ºC overnight. One hour before and for the duration of the assay, media was exchanged for bicarbonate free seahorse assay media composed of DMEM Base (8.3g per liter of medium, Sigma) supplemented with 10mM glucose, 2mM Glutamax-1 100x (Gibco), 1mM sodium pyruvate (Gibco) in MQ H20, pH adjusted to 7.4. Cell plates were left to equilibrate for 1hr at 37ºC in a non-CO2 incubator.

During the assay four basal oxygen consumption rate (OCR, reported in pmol/min) measurements were taken. Subsequently, the mitochondrial membrane modulators oligomycin (1 µM), followed by FCCP (1 µM) and lastly antimycin A/rotenone (both 1 µM) were injected (four measuring cycles for each). Basal respiration, ATP production, maximal respiration, spare respiratory capacity and proton leak are defined by the differences between average measurements 4-16, 4-8, 12-16, 4-9 and 8-16, respectively. Non-mitochondrial respiration is defined by average measurement 16.

For normalization, cells were lysed with 30µl 1x RIPA buffer (Sigma) per well and total protein was quantified with the Pierce™ BCA Protein Assay Kit (Thermo Scientific) according to the manufacturer’s instructions. Absorbance was measured at 562nm on a GloMax®-Multi Microplate Multimode Reader.

**Glycolysis Stress test**

The Seahorse XF Glycolysis Stress Test was performed following the manufacturer’s instructions (Seahorse Bioscience, Billerica, MA, USA). Briefly, cells were seeded on Seahorse XFp Cell Culture Miniplates at 7500 cells per well, and allowed to attach and proliferate in a controlled cell incubator. For the assay, the media was exchanged for a glycolysis stress media composed of DMEM Base (8.3g per liter of medium, Sigma) supplemented with 2mM Glutamax-1 100x (Gibco), 1mM sodium pyruvate (Gibco) in MQ H20, pH adjusted to 7.4. During the assay the extracellular acidification rate (ECAR, reported in mpH/min) measurements were taken. Subsequently, glucose (10 mM), followed by oligomycin (1 µM) and 2-deoxy-glucose (2-DG, 100 mM) were injected (four measuring cycles for each). Basal glycolysis, glycolytic capacity and glycolytic reserve were calculated by the differences between average measurements 4-8, 12-16, 8-12, respectively. Non-glycolytic acidification was defined by average measurement 16.

For normalization, cells were lysed with 30µl 1x RIPA buffer (Sigma) per well and total protein was quantified with the Pierce™ BCA Protein Assay Kit (Thermo Scientific) according to the manufacturer’s instructions. Absorbance was measured at 562nm on a GloMax®-Multi Microplate Multimode Reader

**Flow cytometry**

The following mAb conjugated with FITC/Alexa 488, PE, PE-Cy5 or APC/Alexa 647 were used for flow cytometric analysis:

| Target molecule | Clone | Manufacturer |
| --- | --- | --- |
| CD3 | HIT3a | BioLegend |
| CD4 | OKT4 |  |
| CD25 | BC96 |  |
| NKG2D/CD314 | 1D11 |  |
| NKp30/CD337 | P30-15 |  |
| CD107a/LAMP-1 | H4A3 |  |
| CD90/Thy1 | 5E10 |  |
| CD14 | 47-3D6 | ImmunoStep |
| CD163 | GH/S1 | BD Biosciences |
| CD8 | RPA-T8 |  |
| CD1a | HI149 |  |
| IFN gamma | B27 |  |
| Granzyme B | GrA-11 | ImmunoTools |
| Perforin | delta G9 |  |
| CD56 | AF12-7H3 | MiltenyiBiotec |

Table S1

**PCR analysis**

Real-time PCR was performed with the following Taq-man assays:

| Gene name | Reference |
| --- | --- |
| MICA | Hs00792195_m1 |
| MICB | Hs00792956_m1 |
| ULBP1 | Hs00360941_m1 |
| ULBP2 | Hs00607609_m1 |
| B6H7 | Hs02340611_m1 |
| CCL2 | Hs00234140_m1 |
| PTGS2 (COX2) | Hs00153133_m1 |
| LGALS1 | Hs00355202_m1 |
| TLR3 | Hs00152833_m1 |
| TLR4 | Hs00152939_m1 |
| IL6 | Hs00985639_m1 |
| CXCL12 | Hs00171022_m1 |
| CCL5 | Hs00174575_m1 |
| CXCL10 | Hs01124251_g1 |
| MMP2 | Hs01548727_m1 |
| VEGFA | Hs00900055_m1 |
| TGFB1 | Hs00998133_m1 |

Table S2

**Supplementary results**


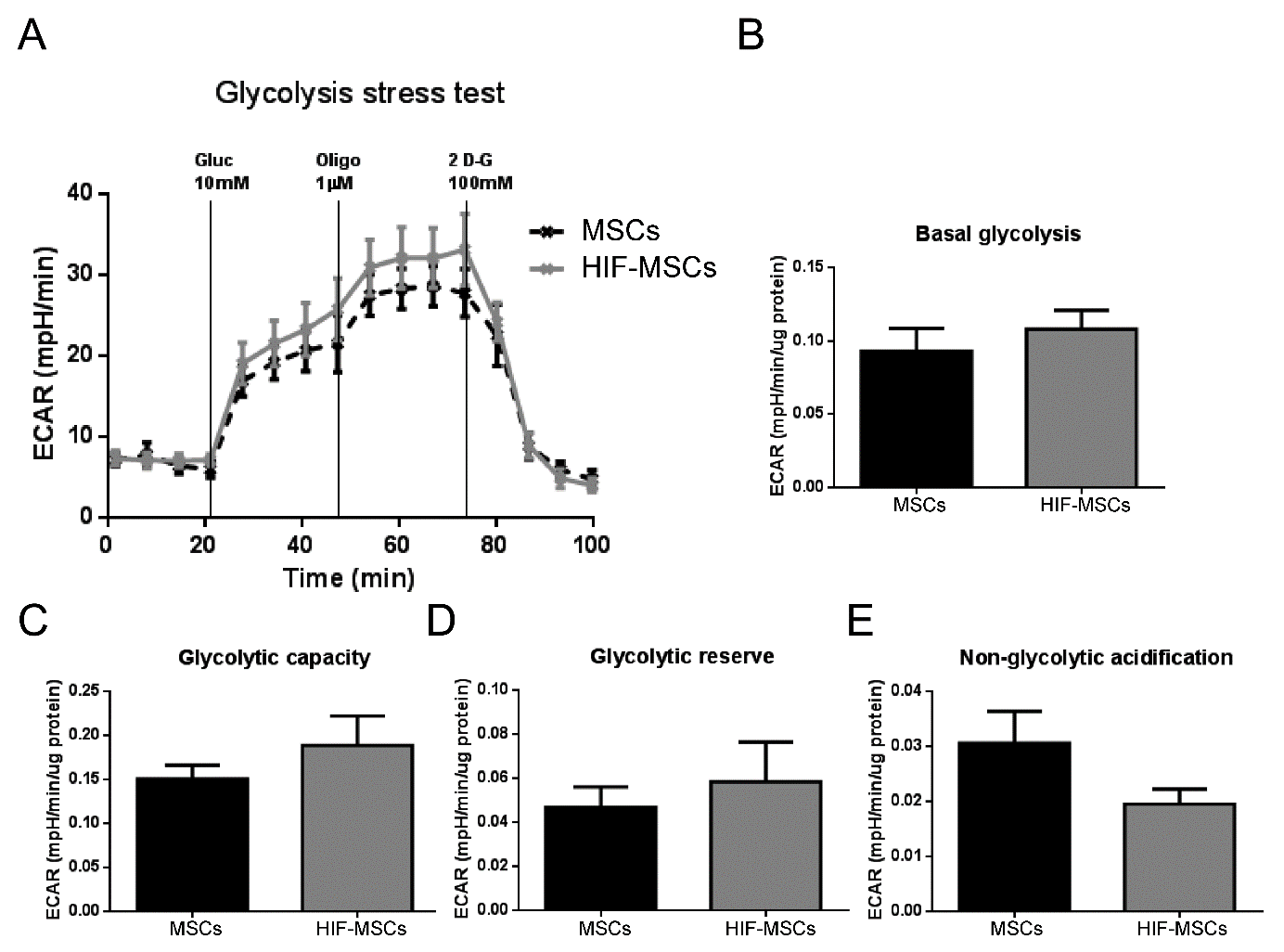


**Figure S1**. HIF -1 alpha increases the glycolytic activity of MSCs. A) Extracellular Acidification Rate (ECAR) response to Glucose (10 mM), Oligomycin (1 µM) and the glycolisis inhibitor 2-deoxyglucose (2-DG, 100 mM) on MSCs (black) and HIF-MSCs (grey). The key parameters of glycolytic flux were also measured: Basal glycolysis (B), glycolytic capacity (C), glycolytic reserve (D) and non-glycolytic acidification (E). Data were normalized against the total amount of protein. Data are represented as mean ± SD. The experiment was repeated 6 times.

|  |  | HIF-MSC vs GFP-MSC | | MSC Hx vs MSC Nx | |
| --- | --- | --- | --- | --- | --- |
| GO_id | **Term** | **Candidates contained** | **p-value** | **Candidates contained** | **p-value** |
| GO:0001666 | Response to hypoxia | 28 (9.3%) | 3.19e-06 | 39 (13.0%) | 4.20e-21 |
| GO:0036293 | Response to decreased oxygen levels | 28 (9.1%) | 5.63e-06 | 39 (12.6%) | 1.20e-20 |
| GO:0061621 | Canonical glycolysis | 8 (30.8%) | 2.10e-06 | 14 (53.8%) | 6.90e-18 |
| GO:0006096 | Glycolytic process | 12 (17.9%) | 3.54e-06 | 16 (23.9%) | 1.40e-13 |
| GO:0006757 | ATP generation from ADP | 12 (17.9%) | 3.54e-06 | 16 (23.5%) | 1.80e-13 |
| GO:0071456 | Cellular response to hypoxia | 21 (15.2%) | 1.74e-08 | 21 (15.2%) | 2.90e-13 |
| GO:0036294 | Cellular response to decreased oxygen levels | 21 (14.5%) | 4.22e-08 | 21 (14.5%) | 7.90e-13 |
| GO:0071453 | Cellular response to oxygen levels | 22 (14.3%) | 2.60e-08 | 21 (13.6%) | 2.60e-12 |
| GO:0031982 | Vesicle | 169 (4.2%) | 0.50e-02 | 110 (2.8%) | 4.10e-05 |
| GO:0001568 | Blood vessel development | 33 (5.7%) | 0.54e-02 | 26 (4.5%) | 7.80e-05 |
| GO:0048514 | Blood vessel morphogenesis | 25 (5.1%) | 0.48e-01 | 22 (4.5%) | 0.30e-03 |
| GO:0005978 | Glycogen biosynthetic process | 5 (9.3%) | 0.42e-01 | 6 (11.1%) | 0.62e-03 |
| GO:0005977 | Glycogen metabolic process | 8 (9.5%) | 0.97e-02 | 7 (8.3%) | 0.13e-02 |
| GO:0097411 | Hypoxia-inducible factor-1alpha signaling pathway | 3 (75%) | 0.17e-03 | 2 (50.0%) | 0.22e-02 |
| GO:0005980 | Glycogen catabolic process | 6 (21.4%) | 0.37e-03 | 3 (10.7%) | 0.17e-01 |

**Table S3.** Gene Ontology (GO) of differentially expressed pathways in mesenchymal stem cells overexpressing HIF-1alpha (HIF-MSC) or submitted to hypoxia conditions (MSC Hx). Gene expression was determined by microarray in control transduced MSC (GFP-MSC), HIF-MSC, normoxia-cultured MSCs (MSC Nx) and hypoxia cultured MSCs (MSC Hx) and compared using consensuspathdb software. A list of genes overexpressed in HIF-MSC vs MSC and in MSC Hx vs MSC Nx was submitted to Gene Ontology (GO) analysis. Enrichment results are shown as a number of gene candidates annotated in each pathway showing the resulting percentage of genes annotated in each set together with p-values for each comparison.

**References**

1. Sen S, Domingues CC, Rouphael C, Chou C, Kim C, Yadava N. Genetic modification of human mesenchymal stem cells helps to reduce adiposity and improve glucose tolerance in an obese diabetic mouse model. Stem Cell Research & Therapy. BioMed Central; 2015;6:242.
